# Life expectancy with chronic kidney disease: an educational review

**DOI:** 10.1007/s00467-016-3383-8

**Published:** 2016-04-26

**Authors:** Guy H. Neild

**Affiliations:** UCL Centre for Nephrology, University College, London, NW3 2PF UK

**Keywords:** Adolescent, Chronic kidney disease, Progressive renal failure, Life expectancy, CAKUT, End-stage kidney disease

## Abstract

Can renal prognosis and life expectancy be accurately predicted? Increasingly, the answer is yes. The natural history of different forms of renal disease is becoming clearer; the degree of reduction in glomerular filtration rate (GFR) and the magnitude of proteinuria are strong predictors of renal outcome. Actuarial data on life expectancy from the start of renal replacement therapy are available from renal registries such as the U.S. Renal Data System (USRDS), and the UK Renal Registry. Recently, similar data have become available for patients with chronic kidney disease. Data collected from a large population-based registry in Alberta, Canada and stratified for different levels of estimated GFR (eGFR) have shown that the reduction in life expectancy with kidney failure is not a uremic event associated with starting dialysis but a continuous process that is evident from an eGFR of ≤60 ml/min. Nevertheless, despite the poor prognosis of the last stages of renal failure, progress in the treatment and management of these patients and, in particular, of their cardiovascular risk factors continues to improve long-term outcome.

## Introduction

How much do we know about renal prognosis and life expectancy in adolescents with chronic kidney disease (CKD)? If one sees a new patient, a 19-year-old youth with a serum creatinine level of 200 μmol/l, can one predict his likely renal prognosis and his life expectancy? The answer is yes, and this is frequently done when the question is posed in a medico-legal context; however, is the answer accurate?

We know that life expectancy is much reduced with end-stage renal failure—but what about the different degrees or stages of renal failure? For this review I have searched the adult and paediatric literature for papers cited in PubMed and Google Scholar that might contain data on life expectancy with CKD, or for series that have followed patients with CKD from childhood to end-stage kidney disease (ESKD) and through to renal replacement therapy (RRT). I summarise the evidence on the prediction of renal prognosis, describe important new data from Canada that for the first time looks at life expectancy with different stages of CKD and cite the U.S. Renal Data System (USRDS) and UK renal registries that report annual data regarding life expectancy with RRT.

## Predicting renal outcome

To predict renal outcome I first make a number of assumptions. On the balance of probabilities (medico-legal language for a >50 % chance), at this age (19 years) the patient will have some form of renal dysplasia that would fall under the general heading of congenital anomalies of the kidney and urinary tract (CAKUT)—or some other congenital disease that might be tubular. If my history and examination make both of these possibilities unlikely, then further investigation is required which might include a biopsy.

If the patient has no proteinuria (protein creatinine ratio <50 mg/mmol), then the renal function should be currently stable. Renal deterioration will not occur until there is increasing proteinuria [1–5]. The exception to this would be a pure tubular disease, and I am assuming that this disease will have been picked up during the history, examination and other basic investigations.

Patients with inexorably progressive renal failure tend to deteriorate at a rate proportional to their proteinuria [6], but generally speaking the more proteinuria, the more the rate of progression can be slowed by angiotensin converting enzyme inhibitors (ACEIs) and good control of blood pressure [2, 7–9].

Patients with small asymmetric kidneys (renal hypodysplasia—often described in the UK as reflux nephropathy) tend to deteriorate at the slowest rates, and this is rarely greater than an estimated glomerular filtration ration (eGFR) of 3–4 ml/min/1.73 m^2^/year [3, 7]. Studies by of our own group have shown that controlling blood pressure and reducing proteinuria with an ACEI should reduce the rate of loss down to around 1.5 ml/min/1.73 m^2^/year [2, 7].

Assuming that the 19-year-old patient with a serum creatinine level of 200 μmol/l has an eGFR of 35 ml/min/1.73 m^2^ and that he will need dialysis when his eGFR is around 10 ml/min/1.73 m^2^, then he should reach ESRD in approximately 17 years [(35 − 10) divided by 1.5 years]. If he were to lose function at the faster rate of 3 ml/min/year, this would be 8.3 years.

## Life expectancy with CKD

Life expectancy tables for people with CKD have been created from a large population-based registry in Alberta, Canada and stratified for different levels of eGFR [10]. Data are calculated for men and women from 30 years of age to age 85 years by their levels of kidney function as defined by eGFRs of ≥60, 45–59, 30–44 and 15–29 ml/min/1.73 m^2^ (see Table 1) [10]. These data show that life expectancy is progressively reduced with each age band of worse renal function.

**Table 1:** Chronic kidney disease and life expectancy^a^

Gender	Age group (year)	Kidney function (in ml/min/1.73 m^2^)			
		eGFR ≥60	eGFR 45–59	eGFR 30–44	eGFR 15–29
Male	30	39.1 (38.9–39.2)	28.4 (25.1–31.7)	20.1 (16.5–23.7)	15.3 (11.0–19.5)
	35	34.7 (34.6–34.9)	28.0 (26.3–29.8)	16.3 (13.3–19.2)	13.8 (11.0–16.7)
	40	30.5 (30.3–30.6)	24.5 (23.3–25.8)	14.5 (12.3–16.8)	10.4 (8.1–12.7)
	45	26.2 (26.1–26.4)	21.3 (20.4–22.2)	12.5 (10.9–14.2)	8.8 (7.1–10.5)
	50	22.3 (22.2–22.4)	18.3 (17.7–19.0)	10.6 (9.5–11.7)	7.4 (6.1–8.7)
	55	18.6 (18.5–18.7)	16.0 (15.5–16.5)	8.7 (7.9–9.5)	6.6 (5.6–7.6)
	60	15.1 (15.0–15.2)	13.6 (13.2–13.9)	7.8 (7.3–8.4)	5.6 (4.8–6.3)
	65	11.9 (11.8–12.0)	10.9 (10.7–11.2)	6.6 (6.2–7.0)	4.6 (4.2–5.1)
	70	9.0 (9.0–9.1)	8.4 (8.3–8.6)	5.9 (5.7–6.2)	3.9 (3.6–4.2)
	75	6.7 (6.6–6.7)	6.2 (6.0–6.3)	4.7 (4.5–4.9)	3.1 (2.9–3.3)
	80	4.6 (4.6–4.7)	4.3 (4.2–4.4)	3.4 (3.3–3.4)	2.5 (2.5–2.6)
	85	2.7 (2.5–2.8)	2.3 (2.2–2.5)	1.8 (1.6–2.0)	1.4 (1.2–1.7)
Female	30	43.8 (43.7–44.0)	33.6 (31.0–36.2)	21.4 (17.3–25.5)	12.7 (7.4–18.0)
	35	39.2 (39.0–39.3)	30.8 (28.9–32.8)	17.6 (14.0–21.2)	13.1 (10.1–16.0)
	40	34.6 (34.5–34.7)	28.7 (27.5–29.9)	16.5 (14.0–19.0)	9.1 (6.6–11.6)
	45	30.2 (30.1–30.4)	25.4 (24.5–26.3)	14.9 (13.0–16.7)	7.4 (5.6–9.3)
	50	26.0 (25.9–26.2)	22.3 (21.7–22.9)	13.2 (11.8–14.5)	7.4 (5.9–8.8)
	55	22.0 (21.9–22.1)	19.1 (18.6–19.6)	11.3 (10.3–12.3)	6.7 (5.6–7.8)
	60	18.2 (18.1–18.3)	16.5 (16.1–16.8)	10.6 (9.9–11.2)	6.2 (5.4–7.0)
	65	14.6 (14.5–14.7)	13.4 (13.1–13.6)	9.4 (8.9–9.9)	4.7 (4.2–5.2)
	70	11.3 (11.2–11.4)	10.5 (10.4–10.7)	7.9 (7.6–8.2)	4.1 (3.8–4.5)
	75	8.4 (8.3–8.5)	7.9 (7.8–8.0)	6.0 (5.9–6.2)	3.9 (3.6–4.1)
	80	5.6 (5.5–5.7)	5.3 (5.2–5.4)	4.5 (4.4–4.6)	3.1 (3.0–3.2)
	85	3.0 (2.9–3.1)	2.8 (2.7–2.9)	2.2 (2.0–2.3)	1.6 (1.4–1.8)

Values in table are presented as the mean life expectancy (in years), with the 95 % confidence interval in parenthesis, according to age, gender and level of estimated glomerular filtration rate (eGFR)

^a^Table is taken from Turin et al. [10] (used with permission)

Assuming our 19-year-old patient will be alive in 11 years, when he reaches 30 (the starting age of the Canadian data), what can be expected? Looking at men age 30–34 years (see Table 1), the life expectancy for those with an eGFR of ≥60 ml/min/1.73 m^2^ is 39.1 years. This is lower than expected and certainly much less than in the UK database. For instance, data from the UK predict that a normal, healthy white male aged 30 years in 2015 has a remaining expected lifetime of 50.7 years [11]. The equivalent figure for the USA suggests that for a 30- to 34-year-old male the expected life expectancy is 45.7 years [12] (see Table 2). The authors of this latter study explain that this difference is attributed to the selective nature of their study cohort, which was limited to individuals who had outpatient serum creatinine measurements as part of routine care. They write that those with an eGFR of >60 ml/min/1.73 m^2^ cannot be considered as a “normal population” as patients having their creatinine measured are likely to be less well than the general population (who would not have a creatinine measure) and therefore have a lower life expectancy.

**Table 2:** Expected remaining lifetime (years) by age, sex, and treatment modality of prevalent dialysis patients, prevalent transplant patients, and the general U.S. population (2012) based on USRDS data and the National Vital Statistics Report^a^

	ESRD patients, 2013				General U.S. population, 2012	
	Dialysis		Transplant		Male	Female
Age	Male	Female	Male	Female		
0–14	24.1	22.4	59.2	61.2	70.7	75.4
15–19	20.9	19.3	46.8	48.6	59.7	64.4
20–24	18.1	16.5	42.5	44.2	55.0	59.5
25–29	15.8	14.3	38.6	40.2	50.3	54.6
30–34	14.1	13.0	34.7	36.4	45.7	49.7
35–39	12.5	11.7	30.8	32.4	41.0	45.0
40–44	10.8	10.3	26.9	28.6	36.4	40.3
45–49	9.1	8.8	23.2	24.8	31.9	35.6
50–54	7.7	7.7	19.8	21.3	27.7	31.1
55–59	6.5	6.6	16.6	18.1	23.7	26.8
60–64	5.5	5.7	13.8	15.2	19.8	22.6
65–69	4.5	4.8	11.4	12.7	16.2	18.5
70–74	3.8	4.0	9.4	10.4	12.8	14.7
75–79	3.2	3.5	7.7^b^	8.6^b^	9.8	11.3
80–84	2.6	2.9			7.1	8.4
85+	2.1	2.4			7.9	5.8

The data reported here have been supplied by the United States Renal Data System (USRDS). The interpretation and reporting of these data are the responsibility of the author(s) and in no way should be seen as an official policy or interpretation of the U.S. government

ESRD, End-stage renal disease

^a^Data Sources are Table H.13 in the 2015 USRDS annual data report [12], special analyses in the USRDS United States Renal Data System database, National Vital Statistics Report (2013, vol 2, chapter 6, Table 6.4). Table 7 . Life expectancy at selected ages, by race, Hispanic origin, race for non-Hispanic population, and sex: United States, 2012 (2015). Expected remaining lifetimes (years) of the general U.S. population and of period prevalent dialysis and transplant patients

^b^Cell values combine ages 75+

From Table 1 it can be seen that for the first three age groups (30–34, 35–39, 40–44 years), life expectancy falls by approximately 20 % with an eGFR of 45–59 ml/min/1.73 m^2^, by approximately 50 % with an eGFR of 30–44  ml/min/1.73 m^2^ and by approximately 65 % with an eGFR of 15–29 ml/min/1.73 m^2^, when compared with those with an eGFR of ≥60 ml/min/1.73 m^2^ (note: these figures are calculated from the first three age groups, i.e. 30, 35 and 40 years, respectively). Thus, the GFR of our patient now age 30 would be approximately 19 ml/min/1.73 m^2^ (eGFR decline of 1.5 ml/min/1.73 m^2^) and that at this level of function his life expectancy is reduced by 70 % from 50.6 to 15 years.

The excess mortality associated with renal failure is due principally to the increased risk of cardiovascular disease. An investigation of the causes of death associated with CKD in Alberta revealed that the major cause of death was cardiovascular (including an increase in heart failure and valvular disease). The unadjusted proportion of patients who died from cardiovascular disease increased with decreasing eGFR [21, 37, 41, and 44 % of patients with an eGFR of ≥60 (with proteinuria), 45–59.9, 30–44.9, and 15–29.9 ml/min/1.73 m^2^, respectively]. The proportion of deaths from infection also increased but not those from cancer [13].

In a separate review using meta-analysis to examine the influence of both reduced eGFR and albuminuria on cardiovascular mortality the authors found that both lower eGFR (<60 ml/min/1.73 m^2^) and higher albumin/creatinine ratio (ACR ≥10 mg/g) were independent predictors of mortality risk in the general population [14]. Adjusted hazard ratios (HRs) for all-cause mortality at eGFRs of 60, 45 and 15 ml/min/1.73 m^2^ (vs. 95 ml/min/1.73 m^2^) were 1.18 [95 % confidence interval (CI) 1.05–1.32], 1.57 (95 % CI 1.39–1.78) and 3.14 (95 % CI 2.39–4.13), respectively. The ACR was associated with mortality risk linearly on the log-log scale without threshold effects. Adjusted HRs for all-cause mortality at ACRs of 10, 30, and 300 mg/g (vs. 5 mg/g) were 1.20 (1.15–1.26), 1.63 (1.50–1.77) and 2.22 (1.97–2.51), respectively. These data are derived from populations a higher mean age, but age was not an independent variable.

Thus, our patient, aged 19–36, even with an eGFR of approximately 45 ml/min/1.73 m^2^, has an increased risk of dying of around 57 % [risk ratio (RR) 1.57] compared with an eGFR of 95 ml/min/1.73 m^2^; similarly, with a ACR of 30 mg/g, our patient has an increased risk of dying of around 63 % (RR 1.63) compared with ACR of 5 mg/g [14]. These figures correlate with life expectancy tables [10] in which a 30-year male with an eGFR of 30–44  ml/min/1.73 m^2^ has a life expectancy reduced by approximately 50 % compared with a similar patient with an eGFR of ≥60  ml/min/1.73 m^2^.

To this equation we should also consider modification of life expectancy by such factors as race, gender and socio-economic status [15, 16], as well as control of blood pressure and hyperlipidemia [17]. All of these factors are being studied in the ongoing Chronic Kidney Disease in Children (CKiD) Study.

## Predicting life expectancy at end-stage

If our patient is well looked after for the next 17 years, I will assume that he will not die before he reaches ESRD at the age of 36 (age 19 + 17 years at a GFR decline rate of 1.5 ml/min/1.73 m^2^/year). However, we now know that this assumption cannot be made. As we have seen from the Canadian data, even at age 19 years with a GFR of 35 ml/min/1.73 m^2^, we can extrapolate that his life expectancy is reduced by around 50 %. For a UK male aged 19 years, a life expectancy of 61.4 years [11] is reduced to 30 years (age 49 years) [10].

Assuming that our patient would be around 36 years of age when end-stage renal failure is reached, then one can use two sources of actuarial information regarding future life expectancy:-

1. The USRDS Annual Report’s chapter on mortality and survival has actuarial tables which show data in 5-year age bands [12] (Table 2). Thus, at 36 years of age, our patient falls into the age band 35–39 years. This shows us that a normal U.S. male of this age group can expect to live a further 41 years. The same age group will live a further 12.5 years on dialysis and 30.8 years after a successful transplant. Of course, in reality, RRT life will tend to be a mixture of the two modes.
2. The UK Renal Registry annual report chapter on survival also has actuarial data in 5-year age bands [18]. However, these show that the median life expectancy for patients starting RRT at the 90-day time point and for this age group (35–39 years) is a further 13.5 years (dialysis and transplant combined).
3. In comparison, the Canadian data show that at age 35 years with an eGFR of 15–29 ml/min/1.73 m^2^, the remaining life expectancy is +13.8 years [10].

## Trends in life expectancy

**Fig. 1:**
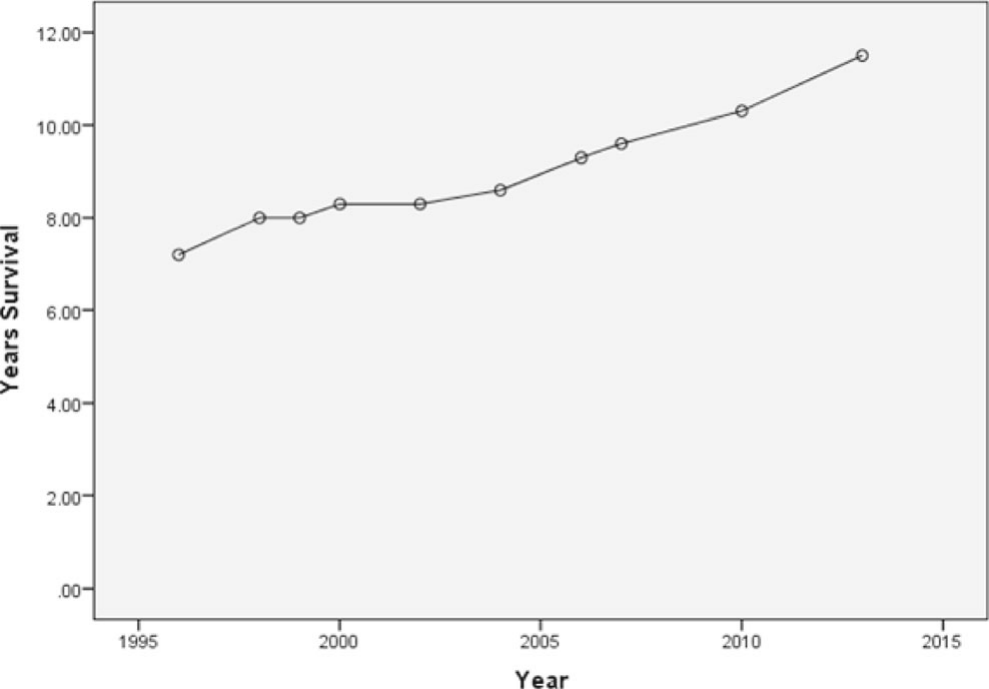
Expected remaining lifetime (years) on dialysis for a 36-year-old man 1996–2013. The data in this figure are taken from on-line archives of United States Renal Data System (USRDS) 1996–2014[12] The interpretation and reporting of these data are the responsibility of the author and in no way should be seen as an official policy or interpretation of the U.S. government

A review of annual reports from the USRDS in the period 1996–2013 reveals that the life expectancy for a 36-year-old man on haemodialysis has improved steadily and linearly from 7.2 years in 1996 to 11.5 years in 2013 (see Fig. 1). Thus, one can anticipate that our current projections of life expectancy probably err on the pessimistic side of reality. This is supported by a detailed analysis of paediatric outcome over the period 1990–2010 [19].

## Summary and conclusions

We can now predict renal outcome and life expectancy with some accuracy, but data sources on life expectancy are few. The new information from Canada on life expectancy with CKD is very important but will need verifying from other parts of the world. We must not forget that collected data are often a decade old before they are analysed and published. While several long-term studies like CKiD [15–17] are running, it is still too early for them to have generated new information on life expectancy. However, trends in outcome continue to improve, suggesting that we can be more optimistic than current data suggest.

## Summary points

1. Life expectancy is reduced for all levels of renal function below an eGFR of 60 ml/min/1.73 m^2^.
2. Actuarial data are now available on life expectancy both for patients with chronic kidney disease and end-stage kidney disease.
3. The increased risk of premature death is principally related to the increase in cardiovascular morbidity.

## Questions (answers are provided following the reference list)

1. Proteinuria predicts progressive renal failure if greater than:

- 50 mg/mmol creatinine (0.5 g/d)
- 100 mg/mmol creatinine (1.0 g/d)
- 150 mg/mmol creatinine
- 200 mg/mmol creatinine

2. Life expectancy is reduced when eGFR falls below:

- 60 ml/min
- 50 ml/min
- 50 ml/min
- 30 ml/min

3. Life expectancy on dialysis in USA has stopped increasing

- Since 2000
- Since 2005
- Since 2010
- Is still increasing

4. The increased relative risk of dying in young patients with CKD is:

- Cardiovascular
- Cancer
- Infection
- None of these

## References

[1] Ardissino G, Testa S, Dacco V, Vigano S, Taioli E, Claris-Appiani A, Procaccio M, Avolio L, Ciofani A, Dello SL, Montini G (2004). Proteinuria as a predictor of disease progression in children with hypodysplastic nephropathy. Data from the Ital Kid Project. Pediatr Nephrol.

[2] Neild GH, Thomson G, Nitsch D, Woolfson RG, Connolly JO, Woodhouse CR (2004). Renal outcome in adults with renal insufficiency and irregular asymmetric kidneys. BMC Nephrol.

[3] Gonzalez CC, Bitsori M, Tullus K (2007). Progression of chronic renal failure in children with dysplastic kidneys. Pediatr Nephrol.

[4] Wingen AM, Fabian-Bach C, Schaefer F, Mehls O (1997). Randomised multicentre study of a low-protein diet on the progression of chronic renal failure in children. Lancet.

[5] Fathallah-Shaykh SA, Flynn JT, Pierce CB, Abraham AG, Blydt-Hansen TD, Massengill SF, Moxey-Mims MM, Warady BA, Furth SL, Wong CS (2015). Progression of pediatric CKD of nonglomerular origin in the CKiD cohort. Clin J Am Soc Nephrol.

[6] Ruggenenti P, Perna A, Mosconi L, Pisoni R, Remuzzi G (1998). Urinary protein excretion rate is the best independent predictor of ESRF in non-diabetic proteinuric chronic nephropathies. “Gruppo Italiano di Studi Epidemiologici in Nefrologia” (GISEN). Kidney Int.

[7] Neild GH (2009). What do we know about chronic renal failure in young adults? II. Adult outcome of pediatric renal disease. Pediatr Nephrol.

[8] The GISEN Group (1997). Randomised placebo-controlled trial of effect of ramipril on decline in glomerular filtration rate and risk of terminal renal failure in proteinuric, non-diabetic nephropathy. Lancet.

[9] Wuhl E, Trivelli A, Picca S, Litwin M, Peco-Antic A, Zurowska A, Testa S, Jankauskiene A, Emre S, Caldas-Afonso A, Anarat A, Niaudet P, Mir S, Bakkaloglu A, Enke B, Montini G, Wingen AM, Sallay P, Jeck N, Berg U, Caliskan S, Wygoda S, Hohbach-Hohenfellner K, Dusek J, Urasinski T, Arbeiter K, Neuhaus T, Gellermann J, Drozdz D, Fischbach M, Moller K, Wigger M, Peruzzi L, Mehls O, Schaefer F (2009). Strict blood-pressure control and progression of renal failure in children. N Engl J Med.

[10] Turin TC, Tonelli M, Manns BJ, Ravani P, Ahmed SB, Hemmelgarn BR (2012). Chronic kidney disease and life expectancy. Nephrol Dial Transplant.

[11] Office of National Statistics (2015). A1.1 2014-based expectation of life (UK). Available at: http://www.ons.gov.uk/ons/search/index.html?newquery=Period+expectations+of+life+%28years%29

[12] United States Renal Data System (2015) Mortality. In: USRDS annual data report: epidemiology of kidney disease in the United States. National Institutes of Health, National Institute of Diabetes and Digestive and Kidney Diseases, Bethesda, chapter 6, vol 2, Table 6.4. Available at: http://www.usrds.org/2015/download/vol2_06_Mortality_15.pdf

[13] Thompson S, James M, Wiebe N, Hemmelgarn B, Manns B, Klarenbach S, Tonelli M (2015). Cause of death in patients with reduced kidney function. J Am Soc Nephrol.

[14] Matsushita K, van der Velde ABC, Woodward M, Levey AS, de Jong PE, Coresh J, Gansevoort RT (2010). Association of estimated glomerular filtration rate and albuminuria with all-cause and cardiovascular mortality in general population cohorts: a collaborative meta-analysis. Lancet.

[15] Wong CJ, Moxey-Mims M, Jerry-Fluker J, Warady BA, Furth SL (2012). CKiD (CKD in children) prospective cohort study: a review of current findings. Am J Kidney Dis.

[16] Hidalgo G, Ng DK, Moxey-Mims M, Minnick ML, Blydt-Hansen T, Warady BA, Furth SL (2013). Association of income level with kidney disease severity and progression among children and adolescents with CKD: a report from the Chronic Kidney Disease in Children (CKiD) Study. Am J Kidney Dis.

[17] Warady BA, Abraham AG, Schwartz GJ, Wong CS, Munoz A, Betoko A, Mitsnefes M, Kaskel F, Greenbaum LA, Mak RH, Flynn J, Moxey-Mims MM, Furth S (2015). Predictors of rapid progression of glomerular and nonglomerular kidney disease in children and adolescents: the chronic kidney disease in children (CKiD) Cohort. Am J Kidney Dis.

[18] Pruthi R, Steenkamp R, Feest T (2014) UK Renal Registry 16th annual report: chapter 8 survival and cause of death of UK adult patients on renal replacement therapy in 2012. Available at: https://www.renalreg.org/wp-content/uploads/2014/09/08-Chap-08.pdf 10.1159/000360027 24662172

[19] Mitsnefes MM, Laskin BL, Dahhou M, Zhang X, Foster BJ (2013). Mortality risk among children initially treated with dialysis for end-stage kidney disease, 1990–2010. JAMA.

